# Prolactin Inhibition Promotes Follicle Recruitment by Increasing *PIKfyve* Expression in Ewes During the Estrus Stage

**DOI:** 10.3390/ani14233541

**Published:** 2024-12-07

**Authors:** Sicong Yue, Chunhui Duan, Yong Wang, Xiangyun Li, Ruochen Yang, Yu Li, Xiangyu Chen, Yueqin Liu, Yingjie Zhang

**Affiliations:** 1 College of Animal Science and Technology, Hebei Agricultural University, Baoding 071000, China; yueyueyuexiaojuan@163.com (S.Y.); duanchh211@126.com (C.D.); lxyun@hebau.edu.cn (X.L.); yangrochen1110@126.com (R.Y.); liyu18730280787@163.com (Y.L.); 2 Institute of Animal Nutrition and Feed, Inner Mongolia Academy of Agricultural & Animal Husbandry Sciences, Hohhot 010031, China; wangyongkeyan0322@126.com; 3 Baoding Livestock Husbandry Workstation, Baoding 071000, China; cxy15630870089@163.com

**Keywords:** prolactin, ewe, *PIKfyve*, follicle recruitment, estrus

## Abstract

Prolactin (PRL) plays a key role in the growth and ovulation of animal follicles, but its impact on follicular recruitment in ewes remains uncertain. In this study, a total of sixteen healthy ewes (Hu sheep, aged 2–3 years, with continuous reproduction and housed separately), matched for parity and weight (52.98 ± 0.96 kg), were randomly assigned to two groups: a control group (C) and a treatment group (T, PRL inhibition). Ovaries were collected in vivo after anesthesia during the estrus stage, and tissue morphology was observed using hematoxylin–eosin (HE) staining. By using RNA sequencing on the ovaries of C and T groups and conducting bioinformatics analysis, the essential genes and pathways involved in the regulation of PRL inhibition were pinpointed. Subcellular localization of key genes in ovarian tissue was determined using a fluorescence in situ hybridization (FISH) assay and immunohistochemistry. The function of key genes was validated using knockout and overexpression techniques. During the estrus phase, we noted a marked rise in the count of large follicles within ovarian tissue following the inhibition of prolactin. In total, 328 differentially expressed genes (DEGs) were detected, with 162 upregulated and 166 downregulated. The results indicated that inhibiting PRL primarily influences follicle recruitment by acting on the target gene *PIKfyve*. Following the inhibition of PRL during the estrus phase, there was an increase in the expression of *PIKfyve*. *PIKfyve* was primarily localized in the ovarian granulosa cells (GCs) and cumulus cells (CCs) in the ovarian tissue of ewes. The overexpression of *PIKfyve* decreased cell apoptosis and enhanced steroid hormone release, whereas knockout of *PIKfyve* had the reverse effect. In conclusion, PRL inhibition promoted follicle recruitment in ewes by upregulating *PIKfyve* during the estrus stage.

## Simple Summary

Prolactin (PRL) is essential for the growth and ovulation of animal follicles, but its impact on follicular recruitment in ewes remains poorly understood. This study identified key genes and pathways influenced by PRL inhibition using RNA sequencing and bioinformatics approaches. Subcellular localization for the key gene in ovarian tissue was observed using a fluorescence in situ hybridization (FISH) assay and immunohistochemistry. The function of these key genes was validated through gene knockout and overexpression experiments. The results indicate that PRL inhibition mainly regulates follicle recruitment through the target gene *PIKfyve*. The subcellular localization of *PIKfyve* in the ovarian tissue of ewes was primarily present in the ovarian granulosa cells (GCs) and cumulus cells (CCs). Overexpression of *PIKfyve* decreased cell apoptosis and enhanced steroid hormone release, whereas knockout of *PIKfyve* had the reverse effect. In conclusion, PRL inhibition facilitates follicle recruitment in ewes by upregulating *PIKfyve* during the estrus phase. These findings offer a fresh perspective on how PRL regulates follicle recruitment during the estrus cycle in ewes.

## 1. Introduction

Annual stocks of sheep in 2021 were 1.47 billion in China [1]. The species has evolved into numerous breeds, which are adapted to fulfill various purposes in different environments [2]. Improvement in reproductive performance is very important in ewes [3]. The estrus cycle represents a fundamental physiological characteristic of placental mammals, initiated by sex hormones following the attainment of sexual maturity [4]; it includes the proestrus, estrus, metestrus, and diestrus stages in ewes [5]. Each cycle encompasses the process of follicular development, involving the activation of primordial follicles, as well as follicular growth, selection, maturation, and ovulation [6]. Ewes experience rapid growth of preantral follicles during the estrus stage and exhibit significant estrus signs, including head-turning, circling, standing, tail fanning, and approaching to ram [7].

Prolactin (PRL) is essential for reproductive processes and is closely associated with estrus and ovulation [5]. As estrus synchronization techniques become increasingly adopted, the role of prolactin inhibition as a regulatory mechanism becomes more critical to improve reproductive efficiency and outcomes. PRL functions through the PRL receptor (PRLR) located on target cells [8]. Halperin et al. found that mice expressing only the short form of *PRLR* display an ovarian phenotype characterized by rapid follicular recruitment, which is subsequently followed by extensive follicular loss, resulting in premature ovarian failure [9]. In addition, the binding of ligands to *PRLR* and its subsequent activation trigger the downstream activation of the canonical Jak2/STAT5 or Jak1/STAT3 pathways, which engage multiple signaling cascades, including phosphoinositide 3-kinase (PI3K)/Akt and Raf/Mek/Erk [10]. Bromocriptine (BCR) is the most common dopamine agonist [11,12] that is capable of effectively reducing the level of prolactin within the body [13] and alleviating hypogonadism, galactorrhea, infertility, amenorrhea, and oligomenorrhea due to elevated serum PRL [14]. Studies have shown that BCR enhances follicular recruitment and embryonic development, resulting in increasing fertilization and pregnancy rates [15], but the underlying mechanism remains unclear.

This study aimed to determine the regulatory role that PRL plays in the estrus stage of ewes. This research employed high-throughput RNA sequencing (RNA-Seq) to clarify the molecular mechanisms involved in PRL inhibition in ewes during the estrus phase. Based on the earlier results, we hypothesize that the inhibition of PRL affects the recruitment of the ovaries by regulating a certain gene in the ovaries of ewes during the estrus period.

## 2. Materials and Methods

### 2.1. Experimental Animals

This research was carried out at Zhihao Livestock Technology Co., Ltd. (longitude 116°03 íE, latitude 37°92 íN, and at an altitude of 18 m) in November and was approved by the Animal Ethics Committee of Hebei Agricultural University (license number 2023156). During the experiment, the ewes had unlimited access to fresh water and were fed twice daily at 7:00 a.m. and 3:00 p.m., following standard farm management practices. Each ewe was housed separately. The ewes’ basic diet is detailed in Table A1. The Hu sheep is a breed that remains in estrus year-round.

### 2.2. Experimental Design

**Figure 1:**
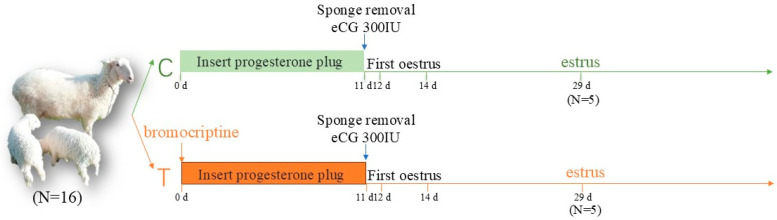
The flowchart of experimental design plan and data collection: Sixteen ewes were randomly divided into two groups, designated as C and T. Ovarian tissues were harvested on 29 d. Estrus synchronization protocol was performed at 0 d [5].

The main activities during the whole experiment are shown schematically in Figure 1. A total of sixteen healthy, non-pregnant Hu sheep aged 2 to 3 years, with an average body weight of 52.98 ± 0.96 kg and matched for parity and weight, were selected and randomly assigned to two groups: control group (C) and treatment group (T, PRL inhibition). We added 2.5 milligrams of BCR to the daily feed of each ewe belonging to the T group, mixed it with water until dissolved, and evenly applied it in a fine mist onto the surface of the feed, starting the treatment at 0 d. After 14 days of induction of estrus, the ewes were again checked for estrus with a vasectomized buck and were found to be in spontaneous estrus. Ovarian tissue was removed and collected from a total of 10 ewes with overt signs of estrus randomly selected from Groups C (PRL concentration = 60.15 ± 1.69 ng mL^−1^) and T (PRL concentration = 54.59 ± 1.50 ng mL^−1^) during estrus. The ovarian tissue was then divided into two parts after being washed with PBS without nuclease. One part was cut into 1 cm^3^ pieces and immediately frozen in liquid nitrogen to extract RNA. The other part was immersed in 10% formaldehyde solution for histological analysis. Three ovarian tissues were collected from both the C group and the T group for transcriptome sequencing.

### 2.3. Tissue Sections Preparation

All ovarian tissue was fixed in 10% formaldehyde solution for 48 h, then trimmed, embedded, and sectioned into 4–7 mm thick slices, followed by staining with hematoxylin and eosin (HE) [16]. After HE staining, sections were mounted with neutral gum. Sections were observed and images were acquired under a Nikon upright fluorescence microscope (Tokyo, Japan).

### 2.4. RNA Extraction, Library Construction, and Sequencing

Total RNA was extracted from the ovarian tissue in the estrus period and granulosa cells (GCs) using the Trizol reagent kit (Invitrogen, Waltham, MA, USA). Next, the enriched mRNA fragments were reverse-transcribed into cDNA [11]. Purified cDNA fragments, after undergoing end repair, were amplified via polymerase chain reaction (PCR). Eventually, the obtained cDNA library was sequenced with Illumina Novaseq 6000 of Guangzhou Genenor Bio-Tech Co., Ltd. (Guangzhou, China). During sequencing, the results can include raw reads that contain low-quality bases or unwanted adapters. To ensure high-quality reads, we used FAST (version 18.0) to filter the reads. Readings containing more than 10% unknown nucleotides (N) or more than 50% low-quality bases (Q value ≤ 20) were eliminated. The DESeq2 method was employed to analyze the discrepancies between experimental groups, and genes with an FDR less than 0.05 and an absolute fold change of at least 2 were considered differentially expressed.

### 2.5. Verification of Differentially Expressed (DE) mRNAs Through qPCR

**Figure 2:**
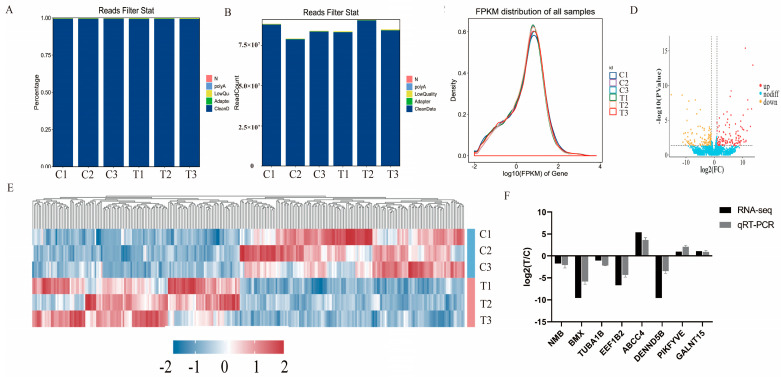
Analysis of RNA-Seq. (**A**) Data preprocessing distribution map among the C and T groups (percentage). (**B**) Data preprocessing distribution map among the C and T groups (numerical value). (**C**) Expression abundance distribution among the C and T groups. (**D**) Volcano plot illustrating the genes with differential expression among the C and T groups. (**E**) Heatmap depicting the genes with differential expression among the C and T groups. (**F**) Validation of selected DEGs was conducted using qRT-PCR. The bar graphs illustrate the mean ± standard error of the mean; C indicates the control group, and T refers to the PRL inhibition group.

To validate the RNA-Seq results, we randomly selected 8 differentially expressed (DE) mRNAs: *NMB*, *BMX*, *TUBA1B*, *EEF1B2*, *ABCC4*, *DENND5B*, *PIKfyve*, and *GALNT15*. As depicted in Figure 2F, the results of qRT-PCR were consistent with those of RNA-Seq, thereby validating the reliability of our RNA-Seq data. The primer sequences are provided in Table 1.

### 2.6. Fluorescence In Situ Hybridization (FISH) to Explore Differential Gene Expression

The tissue was removed and washed with PBS and then immediately placed in an animal in situ hybridization fixative. The tissue specimen was sectioned, dehydrated, and incorporated and embedded in paraffin wax. The paraffin blocks were sliced into 4-μm-thick sections with a microtome, then spread using a spreading machine, and finally subjected to baking. Slices were deparaffinized using 2 replacements of BioDewax and Clear solutions, repaired, and digested. The hybridization probe was added (Table 2 at a concentration of 500 nM. DAPI was added, followed by image collection using fluorescent microscopy [17].

### 2.7. Immunohistochemistry of Paraffin Sections

Following deparaffinization, repair, and digestion, the sections were rinsed with PBS. Antigen retrieval was performed to unmask the antigenic epitope. After the sections were retrieved, primary antibodies (*PIKfyve*, rabbit) and secondary antibodies (IgG) were applied. DAB was subsequently added to visualize the antibody staining. Ethanol dehydration was followed by xylene mounting [18].

### 2.8. Ovine Ovarian GCs Culture and Experimental Design

The subcellular localization of *PIKfyve* in sheep ovarian tissue sections was assessed using a FISH assay and immunohistochemistry. This study found that *PIKfyve* was predominantly localized in the ovarian GCs and follicular membrane cells. To verify the mechanism of action of PRL and *PIKfyve* in the ovaries, sheep ovarian GCs were selected as a cellular model.

Sheep ovaries in good condition were harvested from the Ruili Meat Slaughterhouse in Tang County, Baoding, China. The ovaries were stored in buffered saline containing 1% antibiotics (streptomycin and penicillin, SP) at 37 °C. The ovaries were rinsed with sterile Dulbecco’s Phosphate-Buffered Saline (DPBS), after which GCs were extracted from follicles with diameters ranging from 3 to 7 mm using a syringe. The GCs were subsequently cultured in a basic medium containing 89% DMEM/F12, 10% fetal bovine serum, and 1% SP, as previously used in our laboratory [19,20]. The mixture of follicular fluid and basic medium was centrifuged at 1500× *g* for 15 min to obtain the GCs.

### 2.9. Cell Proliferation and Apoptosis Assay

The GCs were divided into seven groups, with each group consisting of six replicates. Different concentrations of PRL (PRL: ovine, PROSPEC, purity ≥ 99.0%, cyt-240) were added to each group, making the PRL concentrations 0, 4, 10, 20, 30, 40, and 50 ng mL^−1^. This resulted in a final volume of 200 μL in each well, which was then incubated at 37 °C and 5% CO_2_ for 24 h to measure changes in cell proliferation activity. GCs were plated in 96-well plates at a density of 6 × 10^3^ cells per well and incubated for 12 h. The medium of the treatment group was changed after adhesion. Ten μL of Cell Counting Kit-8 (CCK-8) reagent was added to each well and incubated in the dark for 3 h, followed by the determination of the OD value at a wavelength of 450 nm. By plotting based on the OD values, it was revealed that the cell proliferation activity with the addition of 4 ng mL^−1^ of PRL was significantly higher than that of 0, 10, 20, 30, 40, and 50 ng mL^−1^. The optimal PRL concentration was found to be 4 ng mL^−1^. GCs were seeded into 6-well plates at a density of 1 × 10^5^ per well and cultured with basal medium and 4 ng mL^−1^ PRL (basal medium + 4 ng mL^−1^ PRL), respectively. Cell apoptosis was assessed using a Flow Cytometer [20]. All experiments were conducted three times.

### 2.10. Knockout and Overexpression of PIKfyve

**Figure A1:**
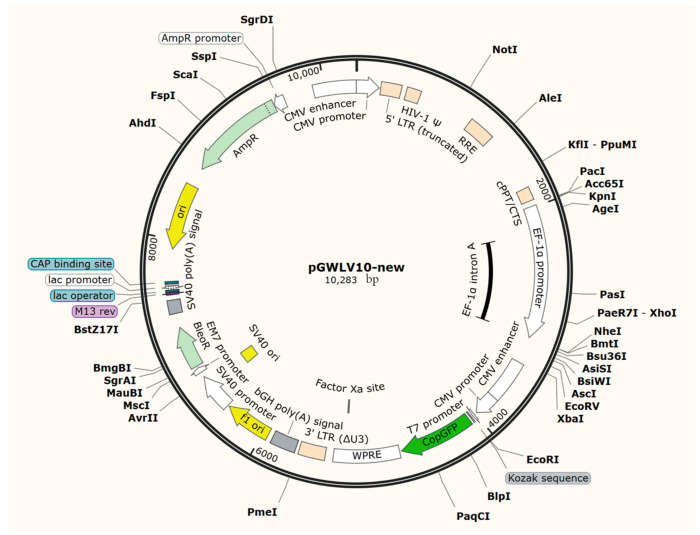
The Plasmid Map of PGWLV10-new.

*PIKfyve* knockout with CRISPR/Cas9: Three small guide RNAs (sgRNAs), each 20–25 base pairs length (as listed in Table 3), were designed to target the *PIKfyve* gene. Chemically synthesized sgRNAs were used to construct CRISPR vectors, which were then transfected into GCs. Next, 3-pass GCs were transfected with either the px458 plasmid (negative control, T-458, containing the empty px458 plasmid as shown in Figure A1), or the *PIKfyve*-knockout recombinant plasmid (T-sg-PIK group). In addition, two other groups included GCs transfected with the recombinant plasmid for *PIKfyve* knockout combined with 4 ng mL^−1^ PRL (T-sg-PIK-PRL group) and GCs treated with 4 ng mL^−1^ PRL (T-PRL group), as well as a general control group of untreated GCs (T group). In each well, 1 × 10^5^ cells were seeded into a 6-well plate for transfection. After incubating the cells overnight at 37 °C, the recombinant plasmid or px458 was transfected into GCs using Lipofectamine 3000 (Invitrogen, Carlsbad, CA, USA) firm successful knockout, and qPCR was used to verify gene expression.

*PIKfyve* overexpression: The plasmids containing lentiviral vectors were provided by Jiangsu Genewiz Biotechy Co., Ltd. (Suzhou, China). GCs were infected with lentiviruses that carried either an empty vector (negative control group: T-pG group) or overexpression sequence targeting *PIKfyve* (T-pG-PIK group). Two other groups were included: lentiviruses containing the overexpression sequence of *PIKfyve*, along with 4 ng mL^−1^ PRL (T-pG-PIK-PRL group), and GCs treated with 4 ng mL^−1^ PRL (T-PRL group), as well as a general untreated GCs group (T group). For the overexpression experiments, 1 × 10^5^ cells were seeded in 6-well plate, and each group’s lentivirus was applied when the cells reached 50% confluence, with an optimal multiplicity of infection (MOI = 350). After 24 h of culturing, the cells were washed with DPBS, and the lentiviral mixture medium was replaced with the basic medium. The GCs were collected at 48 h post-infection to evaluate the impact of overexpression.

### 2.11. E_2_ and P_4_ Hormone Analysis

The concentrations of P_4_ (No. JLC10263) and E_2_ (No. JLC10385) from Shanghai Enzyme-Linked Biotechnology Co. (Shanghai, China) were determined following the manufacturer’s guidelines. The OD was recorded at 450 nm, and a standard curve was established. Based on this standard curve, the hormone concentration in the cell culture medium from the 6-well plate was determined.

### 2.12. Western Blot

Western blot analysis was performed according to established protocols [19,21]. The primary antibodies used were as follows: *PIKfyve* (PIKfyve, 1:1000; 13361-1-AP, Sanying, Wuhan, China) and *GAPDH* (GAPDH, 1:3000; GB15004, Servicebio, Wuhan, China). The goat anti-rabbit IgG (H + L) secondary antibody (1:3000, GB23303, Servicebio) was applied. AlphaView SA software (version 3.4.0, BioTechne, Minneapolis, MN, USA) was used to analyze and quantify the images. The original imagines can be found in Supplementary Materials.

### 2.13. Statistical Analyses

A randomized design was employed, with individual ewes and single-well ovine ovarian GCs as experimental units. Data were analyzed using a one-way ANOVA after assessing independence, normality, and homogeneity with SPSS software (version 26.0). The findings are presented as mean values accompanied by the standard error of the mean (SEM), with a significance level set at *p* < 0.05. Data visualization was carried out utilizing GraphPad Prism 9.0 software along with the ‘ggplot2’ package in R (R-3.5.2).

## 3. Results

### 3.1. HE Staining of Ovarian Tissue in Sheep During Estrus

**Figure 3:**
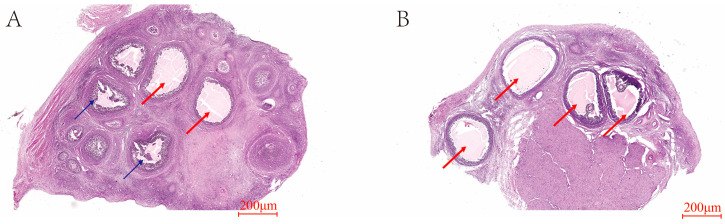
HE staining of ovarian tissue sections in estrus. (**A**) C group. (**B**) T group. The red arrows in the figure represent dominant follicles, and the blue ones represent atretic follicles.

Compared to the C group (Figure 3A), the T group (Figure 3B) exhibited an increase in the number of dominant ovarian follicles, while a decrease in the count of atretic follicles was observed. Ovarian follicles in the T group were oval-shaped and relatively regular.

### 3.2. Transcriptomic Sequencing

After eliminating low-quality reads containing N sequences and adapter sequences, the process yielded a total of 37,777,235,400 raw reads and 38,999,537,400 clean reads. Basic details about the sequencing data are given in Table 4. Figure 2A shows the percentage of clean readings in the series data based on the percentage filtered, while Figure 2B illustrates the number of clean readings based on the filtered values. Figure 2C illustrates the distribution of genes or transcripts across various samples, as determined by FPKM. Samples differed very little in preparation, sequencing, alignment, and quantitation. A volcano plot (Figure 2D) was generated to further investigate the differentially expressed genes (DEGs). In the C group compared to the T group, 166 mRNAs were significantly downregulated, while 162 mRNAs were significantly upregulated (*p* < 0.05, log2 fold change ≥ 1).

### 3.3. Validation of DE mRNAs Using Quantitative Real-Time PCR

To validate our RNA-Seq results, the expression levels of *NMB*, *BMX*, *TUBA1B*, *EEF1B2*, *ABCC4*, *DENND5B*, *PIKfyve*, and *GALNT1* were measured using qPCR. The relative variations of the qRT-PCR results were consistent with those of RNA-Seq, indicating that our RNA-Seq results are both authentic and reliable (Figure 2F).

### 3.4. GO and KEGG Enrichment Analyses of Differential Genes

**Figure 4:**
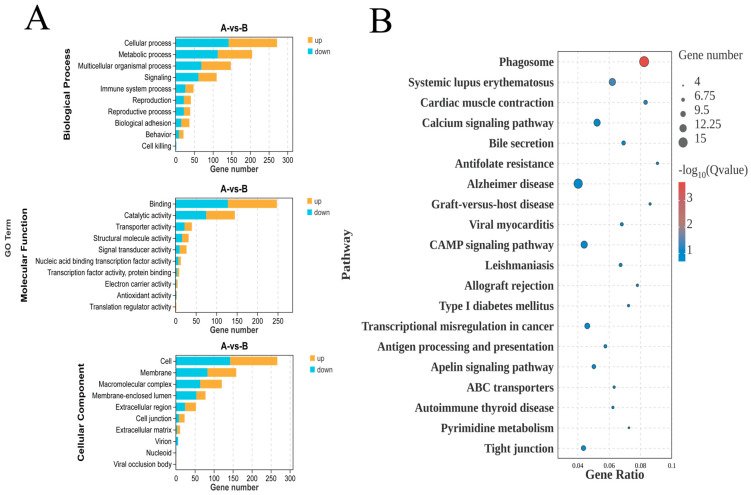
Analyses of GO and KEGG pathway. (**A**) GO functional enrichment analysis was conducted on genes with differential expression, comparing the C and T groups. (**B**) Analysis of KEGG pathways of the top 20 DEGs among the C and T groups.

A total of 60 Gene Ontology (GO) terms were significantly enriched for DEGs, which included biological process (BP), cellular component (CC), and molecular function (MF), with counts of 26, 22, and 12, respectively. Figure 4A illustrates that various GO terms are significantly enriched among the DEGs. GO annotation revealed that the DEGs were involved in several BPs, including cellular processes, single-organism processes, metabolic processes, biological regulation, and the regulation of biological processes. The primary functional categories of differentially expressed genes within CC were the cell, cell part, organelle, organelle part, and membrane, and in MF, they were binding, catalytic activity, and signal transducer activity.

Pathway analyses were conducted using the KEGG pathway database to identify pathways significantly enriched in DEGs. The top 20, based on pathway enrichment analysis, are shown in Figure 4B. This study’s findings revealed that the significant signaling pathways included 246 pathways, such as the phagosome, calcium signaling pathway, cAMP signaling pathway, antigen processing and presentation, ABC transporters, and tight junction pathway. Among these, the phagosome signaling pathway exhibited the most significant difference in the KEGG pathways.

### 3.5. Subcellular Localization of PIKfyve in the Ovaries

**Figure 5:**
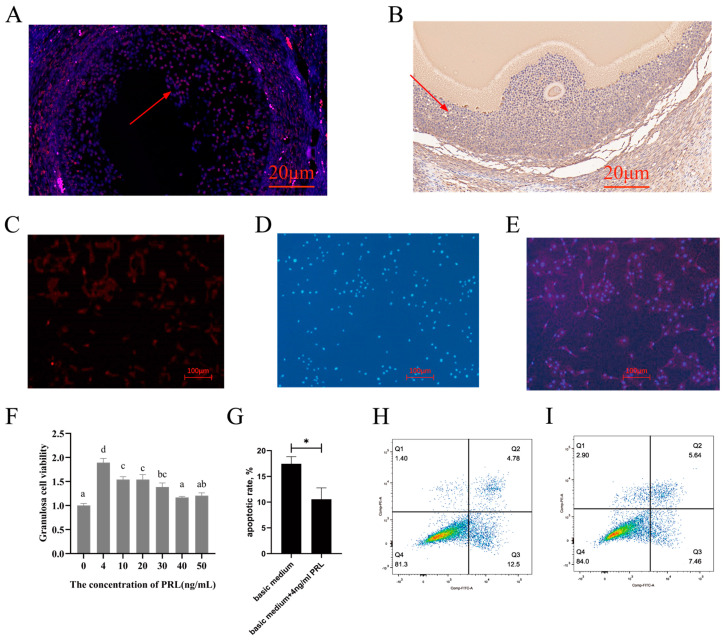
*PIKfyve* mapping, identification of ovine ovarian GCs, and the effects of treatment with different concentrations of PRL. (**A**) FISH of the exploration of *PIKfyve* mapping. The nucleus of DAPI channel is blue, and the positive CY3 channel is red. The fluorescence intensity is strong or weak according to the expression amount. The arrow points to the granulocyte. (**B**) Immunohistochemistry of the exploration of *PIKfyve* mapping. Hematoxylin stains the nucleus of cells blue, and DAB shows positive expression as light brown. The arrow points to the granulocyte. (**C**–**E**) The red markers indicate cells expressing FSHR, while the blue markers indicate nuclei stained with DAPI. The combined images display the superimposition of FSHR labeled with red fluorescence and DAPI labeled with blue fluorescence. (**F**) Effects of the treatment with different concentrations of PRL on viability. The different lowercase letters indicate significant differences (*p* < 0.05). The Y-axis represents the ratio of the proliferation viability of GCs. (**G**) Effects of the treatment with 4 ng mL^−1^ PRL on apoptosis. The calculation of the apoptosis rate is derived from the measurement results of the flow cytometer. The apoptosis rate is equal to the percentage of early withered plus that of late withered. Figure 5G shows the average of three measurements. The asterisk (*) stands for a significant difference. (**H**,**I**) Results from a flow cytometer. X-axis and Y-axis represent PI fluorescence and Annexin-V fluorescence. Q1, Q2, Q3, and Q4 represent dead cells, late withered, early withered, and live cells, respectively.

The subcellular localization of *PIKfyve* in ovarian tissue from sheep was examined using the FISH assay, as shown in Figure 5A, and the immunohistochemistry is shown in Figure 5B. It was found that these genes were primarily present in the ovarian GCs and cumulus cells (CCs).

### 3.6. Immunofluorescence Staining and Cell Proliferation and Apoptosis Assay

The cells expressing Follicle-Stimulating Hormone receptor (*FSHR*) are shown in Figure 5C, marked with a red marker. The cell nuclei were stained with DAPI, as shown in Figure 5D, and are marked in blue. As shown in Figure 5E, the distribution of GCs can be visualized by labeling *FSHR* and nuclei, as *FSHR* proteins were specifically expressed in GCs.

The cell viability with PRL concentration is shown in Figure 5F. Initially, the cell survival rate increased with a rising PRL concentration but then declined (*p* < 0.05). Cell viability showed an increase in the 4 ng mL^−1^, 10 ng mL^−1^, 20 ng mL^−1^, and 30 ng mL^−1^ groups compared to the 0 ng mL^−1^ group (*p* < 0.05). In contrast, there was no difference in viability between the 40 ng mL^−1^ and 50 ng mL^−1^ groups (*p* > 0.05). The apoptosis rates with basic, medium, and 4 ng mL^−1^ PRL are shown in Figure 5G. Dead, late-depleted, early depleted, and live cells are shown in Figure 5H,I. The rate of apoptosis in GCs within the 4 ng mL^−1^ group was lower than that of the control group (*p* < 0.05).

### 3.7. Western Blot Analysis of PIKfyve Overexpression and Knockout Results

**Figure 6:**
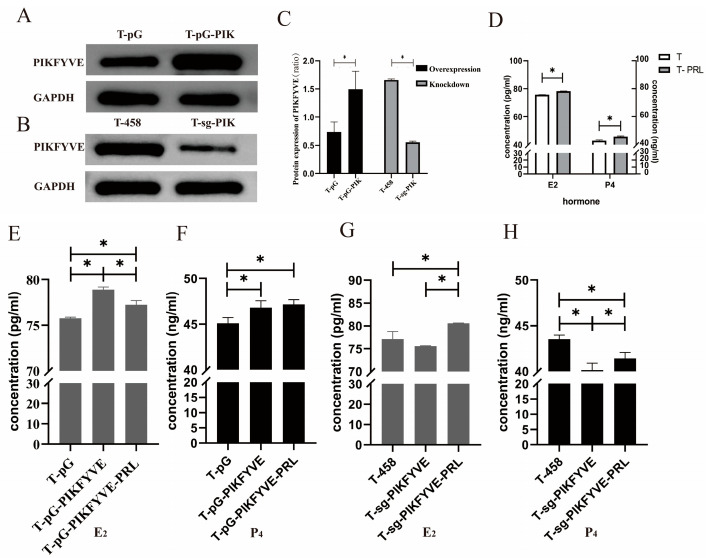
Western blot analysis of *PIKfyve* knockout and overexpression results and the secretion of steroid hormones (E_2_ and P_4_). Each well functions as a replicate, and each experiment was repeated three times, with each replicate being measured three times. The data presented in the figure are expressed as the mean ± SEM. (**A**,**B**) Representative image of the Western blot for *PIKfyve*. (**C**) Western blot quantification of protein content. (**D**) The secretion of steroid hormones (E_2_ and P_4_); T: GCs with basal medium; T-PRL: GCs with 4 ng mL^−1^ PRL; (**E**) The secretion of E_2_ after overexpression of *PIKfyve.* (**F**) The secretion of P_4_ after overexpression of *PIKfyve.* (**G**) The secretion of E_2_ after knockout of *PIKfyve.* (**H**) The secretion of P_4_ after knockout of *PIKfyve*. The asterisk (*) in (**A**–**H**) stands for a significant difference.

Western blot results of the overexpression and knockout of *PIKfyve* are shown in Figure 6A–C. Overexpression *PIKfyve* resulted in an increase in *PIKfyve* protein content, while knockout *PIKfyve* caused a reduction in protein levels (*p* < 0.05).

### 3.8. Effects of the Knockout and Overexpression of the PIKfyve Gene on the Secretion of Steroid Hormones (E_2_ and P_4_)

Figure 6D illustrates the impact of 4 ng mL^−1^ PRL on steroid hormone secretion. In the T-PRL group, the levels of E_2_ and P_4_ increased (*p* < 0.05). Figure 6E–H depict the influence of *PIKfyve* gene overexpression and knockout on steroid hormone secretion. The levels of E_2_ and P_4_ (Figure 6E,F) in the T-pG-PIK group were elevated compared to those in the T-pG group (*p* < 0.05). Compared with the T-pG-PIK group, the concentration of E_2_ in the T-pG-PIK-PRL group was decreased (*p* < 0.05), while there was no difference in the concentration of P_4_ (*p* > 0.05).

The concentrations of P_4_ (Figure 6G,H) in the T-sg-PIK group were lower than those in the T-458 group and T-sg-PIK-PRL group (*p* < 0.05). The concentrations of P_4_ (Figure 6G,H) in the T-sg-PIK-PRL group were lower than those in the T-458 group and higher than those in the T-sg-PIK group (*p* < 0.05). The concentrations of E_2_ in the T-sg-PIK-PRL group were increased in comparison with those in the T-sg-PIK group and T-458 group (*p* < 0.05), and no differences were observed in the T-sg-PIK group and T-458 group (*p* > 0.05).

### 3.9. Expression of Related Genes After the Overexpression and Knockout of PIKfyve

**Figure 7:**
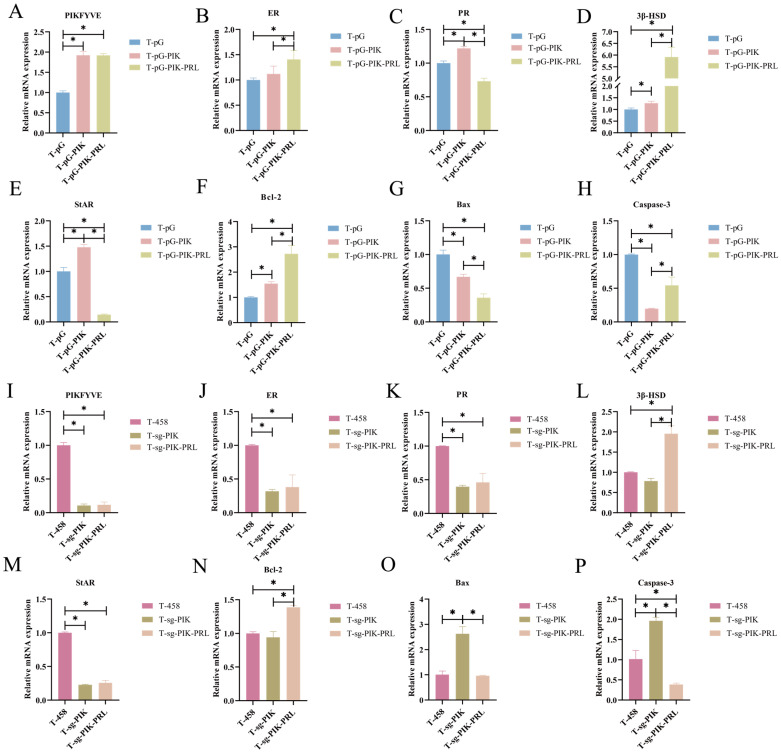
**qPCR of *PIKfyve*, *ER*, *PR*, steroidogenic enzymes, and apoptosis-related genes.** (**A**) qPCR validation of *PIKfyve* after overexpression. (**B**) The relative expression of *ER* after overexpression of *PIKfyve.* (**C**) The relative expression of *PR* after overexpression of *PIKfyve.* (**D**) The relative expression of *3β-HSD* after overexpression of *PIKfyve.* (**E**) The relative expression of *StAR* after overexpression of *PIKfyve.* (**F**) The relative expression of *Bcl-2* after overexpression of *PIKfyve.* (**G**) The relative expression of *Bax* after overexpression of *PIKfyve.* (**H**) The relative expression of *Caspase-3* after overexpression of *PIKfyve.* (**I**) qPCR validation of *PIKfyve* after knockout. (**J**) The relative expression of *ER* after knockout of *PIKfyve.* (**K**) The relative expression of *PR* after knockout of *PIKfyve.* (**L**) The relative expression of *3β-HSD* after knockout of *PIKfyve.* (**M**) The relative expression of *StAR* after knockout of *PIKfyve.* (**N**) The relative expression of *Bcl-2* after knockout of *PIKfyve.* (**O**) The relative expression of *Bax* after knockout of *PIKfyve.* (**P**) The relative expression of *Caspase-3* after knockout of *PIKfyve*. The asterisk (*) in (**A**–**P**) stands for a significant difference.

Figure 7A–H illustrate the gene expression levels following overexpression. *PIKfyve* expression increased (*p* < 0.05) in both the T-pG-PIK group and T-pG-PIK-PRL in comparison with that in the T-pG group, and no difference occurred in the T-pG-PIK group and T-pG-PIK-PRL group (*p* > 0.05). In the T-pG-PIK group, the expression of *PR*, *3β-HSD*, *StAR*, and *Bcl-2* was increased (*p* < 0.05) in comparison with that in the T-pG group, the expression of *Bax* and *Caspase-3* was decreased, and the expression of *ER* had no significant differences (*p* > 0.05). In the T-pG-PIK-PRL group, there was an increase in the expression levels of *ER*, *3β-HSD*, *Bcl-2*, and *Caspase-3* (*p* < 0.05), while the expression of *PR*, *StAR*, and *Bax* showed a decrease when compared to the T-pG-PIK group.

Figure 7I–P illustrate the related gene expression levels following knockout. The level of expression for *PIKfyve* was decreased (*p* < 0.05) in the T-sg-PIK group and T-sg-PIK-PRL in comparison with that in the T-458 group, while no notable difference was observed in the T-sg-PIK group and T-sg-PIK-PRL group (*p* > 0.05). In the T-sg-PIK group, the expression of *ER*, *PR*, and *StAR* was decreased (*p* < 0.05), the expression of *Bax* and *Caspase-3* was increased (*p* < 0.05), and the expression of *3β-HSD* and *Bcl-2* had no significant differences (*p* > 0.05) in comparison with the T-458 group. In the T-sg-PIK-PRL group, the expression of *3β-HSD* and *Bcl-2* was increased (*p* < 0.05), the expression of *Bax* and *Caspase-3* was decreased, and the expression of *ER*, *PR*, and *StAR* had no significant differences (*p* > 0.05) in comparison with the T-sg-PIK group.

**Figure 8:**
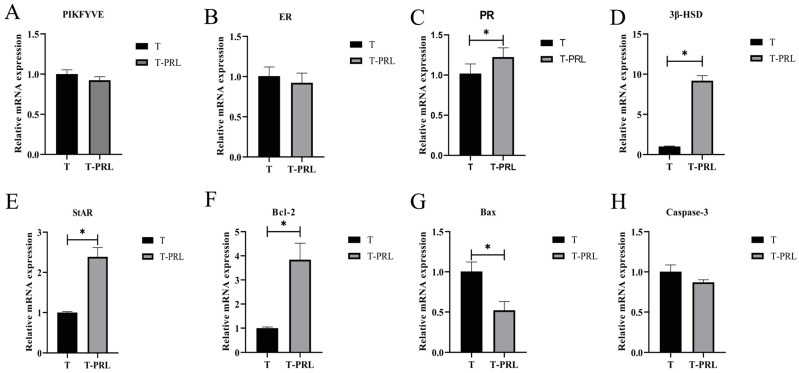
qPCR of *PIKfyve*, *ER*, *PR*, steroidogenic enzymes, and apoptosis-related genes in the T group and T-PRL group. (**A**) qPCR validation of *PIKfyve*. (**B**) The relative expression of *ER*. (**C**) The relative expression of *PR*. (**D**) The relative expression of *3β-HSD*. (**E**) The relative expression of *StAR*. (**F**) The relative expression of *Bcl-2*. (**G**) The relative expression of *Bax*. (**H**) The relative expression of *Caspase-3*; 4 ng mL^−1^ PRL was added to the GCs in the T-PRL group. The asterisk (*) in (**A**–**H**) stands for a significant difference.

Figure 8A–H show the effects of PRL on the expression of related genes; no notable difference was observed in the expression of *PIKfyve*, *ER*, and *Caspase-3* among the T and T-PRL groups (*p* > 0.05). The expression of *PR*, *3β-HSD*, *StAR*, and *Bcl-2* was increased in the T-PRL group in comparison with that in the T group (*p* < 0.05), and the expression of *Bax* decreased (*p* < 0.05) in the T-PRL group in comparison with that in the T group.

## 4. Discussion

### 4.1. The Effect of PRL on Follicular Development

The ovarian tissue undergoes significant functional and morphological changes throughout the estrus cycle. This process includes the development and eventual degeneration of follicles, along with the formation of the corpus luteum, which subsequently undergoes atrophy [22]. PRL has a modulatory role in follicular growth and maturation and in the selection of ovulatory follicles [23], thus promoting oocyte maturation, meiosis, fertilization, and early embryonic development [24]. This study suggests that suppression of PRL increases the number of dominant follicles during the estrus stage. This result aligns with earlier research that demonstrated a gradual reduction in follicle numbers as PRL concentration increased [20]. During the estrous cycle, atresia is the common outcome of follicle development, as most follicles degenerate before they reach maturity [22]. The secretion of PRL has a preovulatory surge that occurs in proestrus [5] concomitantly with the FSH surge [25]. PRL inhibition promotes the secretion of FSH and LH from the pituitary and promotes the reserve of FSH in the pituitary [26,27]. During the estrus cycle, the increase in FSH released by the pituitary gland can effectively reverse atresia [22]. In reaction to stimulation by FSH, a limited number of antral follicles transition into a stage of recruitment for follicle development that relies on gonadotropins [28]. The role of GCs is to facilitate oocyte growth, promote the expansion of cumulus cells in response to FSH, and carry out endocrine functions that aid in follicle development [29]. The introduction of prolactin inhibits ovulation induced by hCG, and this effect is related to the dosage [30]. For example, lower concentrations increased the viability and proliferation of follicles and increased apoptosis in a concentration-dependent manner [31,32]. In addition, GCs may affect follicular development and maturation by blocking the AMPK signaling pathway [33]. Our findings show that GC survival rates increased in the 4 ng mL^−1^, 10 ng mL^−1^, 20 ng mL^−1^, and 30 ng mL^−1^ PRL groups. However, no notable differences were detected in the 40 ng mL^−1^ and 50 ng mL^−1^ groups. Low concentrations of PRL attenuated cytotoxicity and oxidative stress, whereas high concentrations of PRL induced oxidative stress by modulating ROS mitophagy, ultimately leading to increased cytotoxicity [32]. This finding was consistent with previous studies and filled a gap in studies where PRL concentrations ranged from 4 ng mL^−1^ to 50 ng mL^−1^, indicating that a low concentration of PRL may rescue follicular atresia by affecting FSH secretion in estrus.

### 4.2. Effect of PIKfyve on Hormone Secretion and Apoptosis in GCs

In this study, the phagosome pathway was determined to be the most significantly enriched one. Furthermore, *PIKfyve* was highly expressed within the phagosome pathway following PRL inhibition. *PIKfyve* is a crucial lipid kinase in mammals with diverse cellular functions, and its genetic knockout in mice results in preimplantation lethality [34]. The activity of *PIKfyve* is essential for several physiologically significant pathways, including the digestion of autophagic and phagocytic cargo as well as endocytosed receptors [35]. Our study showed that overexpression of *PIKfyve* at an optimal PRL concentration significantly increased the anti-apoptotic ability and hormone secretion of GCs. This suggests that PRL concentration can affect the expression of *PIKfyve*. However, the regulatory mechanism of *PIKfyve* in follicle development in ovarian tissue in ewes has not yet been studied.

This research disclosed that *PIKfyve* was primarily present in ewes ovarian GCs and CCs according to FISH assay and immunohistochemistry. GCs secrete hormones (E_2_, P_4_) that are imperative for follicular development [36]. Our study showed that the overexpression of *PIKfyve* improved steroid hormone production capacity by significantly increasing the levels of E_2_ and P_4_, along with increasing the expression of *PR*, *3β-HSD*, and *StAR*, while the knockout of *PIKfyve* reduced these by decreasing the concentration of P_4_ and the expression of *ER*, *PR*, and *StAR*. *ER* and *PR*, both constituents of the nuclear receptor family, modulate transcriptional activities upon complexation with E_2_, thereby exerting a further impact on antral follicular development and oocyte maturation [5]. In GCs, FSH relies on the CAMP pathway to regulate aromatase activity, while phosphatidylinositol 3-kinase (PI3K) can regulate the action of this enzyme [37]. Inhibitors of PI3K could block the activation of Akt with E_2_, which is dependent on time and dose [38]. Furthermore, the inhibition of *PIKfyve* also triggers a unique lipid biosynthesis and metabolism process by upregulating genes such as FASN and ACACA, leading to a dependence on novel lipid metabolic pathways [39]. PI(4,5)P2, which is synthesized locally by PIP5K in lipid droplets (LDs), plays a role in the transfer of lipids between LDs and the endoplasmic reticulum, thereby influencing ovarian progesterone production by regulating various dynamic activities of LDs [40]. The principal action of *StAR* is to assist in the transport of cholesterol from the outer mitochondrial membrane to the inner mitochondrial membrane, while *3β-HSD* converts pregnenolone produced from cholesterol into progesterone [41]. This means that the overexpression of *PIKfyve* improves the process of delivering cholesterol to mitochondria and steroid enzymes.

GCs and CCs mediate the ovarian follicular microenvironment and are accountable for fostering the growth and development of oocytes, along with the progressive attainment of their developmental competence [42]. Apoptosis and autophagy of GCs regulate the development and atresia of follicles [43]. Our study showed that the overexpression of *PIKfyve* improved anti-apoptotic ability by significantly increasing the expression of *Bcl-2* and decreasing the expression of *Bax* and *Caspase-3*, while the knockout of *PIKfyve* improved apoptosis by increasing the expression of *Bax* and *Caspase-3* in the GCs of ewes. Earlier research indicated that the inhibition of *PIKfyve* can reduce the proliferation and migration of liver cancer cells, lymphoma cells, and fibroblasts [44,45,46], increasing the secretion of exosomes and inducing secretory autophagy [47]. Further, the ablation of *PIKfyve* resulted in cell-cycle arrest [48]. In addition, the inhibition of *PIKfyve* restrained cell proliferation, which preceded the induction of cell death [49]. Under nutrient-rich conditions, *PIKfyve* sequentially facilitates basal autophagic degradation and provides protection against proteotoxic stress through the PI(3,5)P2-dependent reformation of lysosomes from endolysosomes [50]. Activated *PIKfyve* boosts the formation of PtdIns5P-containing autophagosomes, thus driving the upregulation of autophagy [51]. It further participates in follicle atresia by regulating the death of GCs [52]. This further confirms our results in that the *PIKfyve* gene may regulate follicle development by regulating apoptosis and autophagy in ovarian GCs.

## 5. Conclusions

PRL inhibition facilitates the recruitment of ovarian follicles by enhancing the expression of *PIKfyve* during the estrus period. Apoptosis was inhibited and steroid hormone secretion was increased after the overexpression of *PIKfyve*, whereas the knockdown of *PIKfyve* showed the opposite trend in GCs. These findings provided a foundation for understanding the regulatory mechanisms of PRL for follicle recruitment during the estrus stage in ewes.

## Figures and Tables

**Table 1:** List of the primers used in qRT-PCR.

Gene	Sequence (5′-3′)	Size (bp)	Tm (°C)	Accession Number
GAPDH	F: CTGACCTGCCGCCTGGAGAAA R: GTAGAAGAGTGAGTGTCGCTGTT	149	60	NM001190390.1
NMB	F: CCTCCTGCTCTTTGCTCTGC R: RTGTCGTCGTGTCTTCCTGAT	351	60	XM_012098495.3
BMX	F: GGGATGACGAGGTATGTT R: GACTTGCTGCTGTATTTG	113	60	XM_012106269.3
TUBA1B	F: TGGTGAAATGTGACCCTCG R: CCAACCTCCTCATAATCCTT	303	60	XM_027967380.1
EEF1B2	F: AGGTGCTCAACGACTACTT R: GACTCATACTGGGCAAGG	346	60	XM_004004857.4
ABCC4	F: TAAAGAAGCCATCAAAGC R: ATCCGTTCACAGTCAATAA	267	60	XM_027973867.1
DENND5B	F: ATTAGTCGGTCCTGTTGC R: CTGCCATTTCTTTCACGC	345	60	XM_027967785.1
PIKfyve	F: GGACTCCGCTAATGACTT R: TTCTGCCTTCCTTCTGGT	346	60	XM_027965135.1
GALNT15	F: GCCGTGGACCGACATTACTT R: CAGCGATGCGGATCTTGTT	217	60	XM_027961962.1

**Table 2:** List of the probe sequences used in FISH.

Probe Name	Probe Sequence (5′-3′)	Name of the Corresponding Branch Probe	Name of the Corresponding Signal Probe
PIKfyve	CACAGAACATGCTAGGGCACTGATAGG CCCGCATAGTAGAGCCGACAGTAAAA, CATCACTTGTAATCAACCCTTTTTCTTCAT	Branch probe 1	Cy3 signal probe 1

**Table 3:** Primer sequences of sgRNA.

Gene	Sequence (5′-3′)	Accession Number
PIKfyve-sgRNA1	F: caccTGCAGTGTGGTCGACAAAGG	XM_042244119.1
	R: aaacCCTTTGTCGACCACACTGCA	
PIKfyve-sgRNA2	F: caccTCGATCTTCAGTGTTAGCAG	XM_042244119.1
	R: aaacCTGCTAACACTGAAGATCGA	
PIKfyve-sgRNA3	F: caccTTCGATCCAGATAAACACTA	XM_042244119.1
	R: aaacTAGTGTTTATCTGGATCGAA	

**Table 4:** Detailed information on RNA sequencing.

Sample	Raw Data	Clean Data (bp)	AF_Q20 (%)	AF_Q30 (%)	AF_N (%)	AF_GC (%)
C1	13,270,038,300	13,046,615,014	12,716,667,231 (97.47%)	12,191,900,118 (93.45%)	44,836 (0.00%)	6,912,727,439 (52.98%)
C2	11,886,621,600	11,686,989,200	11,409,161,836 (97.62%)	10,959,006,698 (93.77%)	40,448 (0.00%)	6,055,955,078 (51.82%)
C3	12,620,575,500	12,419,223,142	12,107,662,243 (97.49%)	11,606,200,264 (93.45%)	43,592 (0.00%)	6,495,683,032 (52.30%)
T1	12,574,679,100	12,324,486,148	11,916,629,983 (96.69%)	11,328,352,819 (91.92%)	163,212 (0.00%)	6,484,193,767 (52.61%)
T2	13,668,156,900	13,425,744,796	12,983,418,823 (96.71%)	12,319,214,173 (91.76%)	180,880 (0.00%)	6,856,843,493 (51.07%)
T3	12,756,701,400	12,500,983,758	12,049,751,708 (96.39%)	11,403,173,233 (91.22%)	167,307 (0.00%)	6,545,461,954 (52.36%)

**Figure A2:**
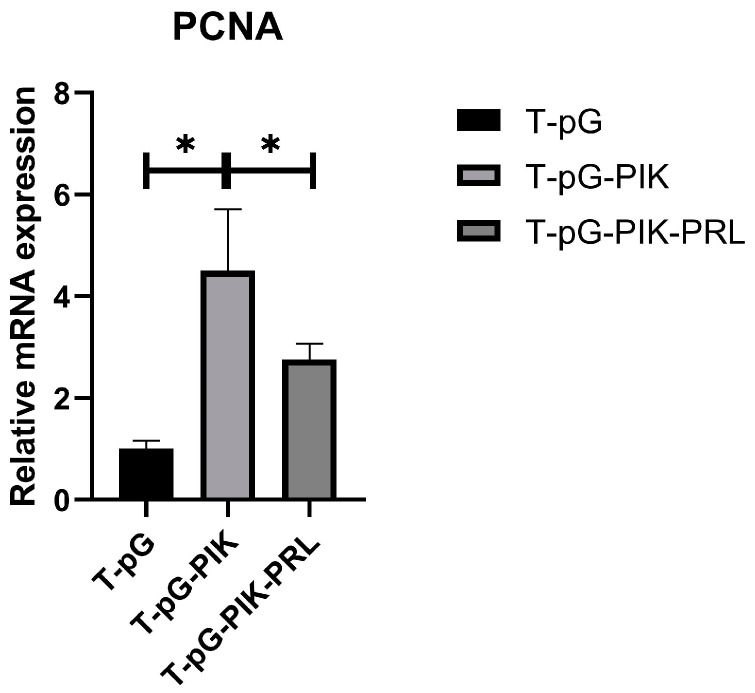
qPCR of *PCNA* in the knockout group. The asterisk (*) stands for a significant difference.

**Figure A3:**
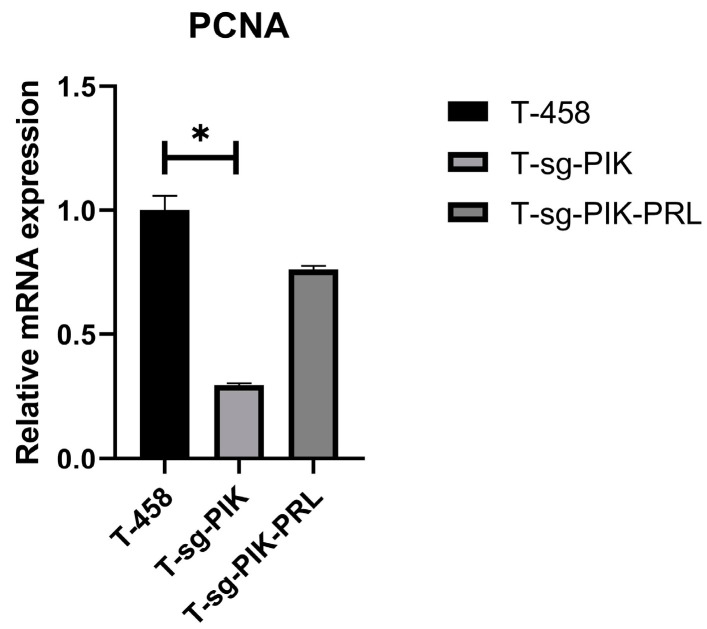
qPCR of *PCNA* in the overexpression group. The asterisk (*) stands for a significant difference.

## Data Availability

All datasets generated and analyzed during the current study are available from the corresponding author (zhangyingjie66@126.com) upon reasonable request.

## Supplementary Materials

The following supporting information can be downloaded at: https://www.mdpi.com/article/10.3390/ani14233541/s1, WB original images.

## Appendix A

**Table A1:** Ingredients and nutrient composition of the basal diet (dry matter basis).

Item	Content (%)
Ingredient	
Hay	42.00
mineral meal	0.40
Soybean meal	12.00
Corn	44.20
CaHPO_4_	0.28
Premix ^1^	0.59
NaCl	0.53
Total	100.00
Nutritional Indicator	
Metabolic Energy, ME/(MJ kg^−1^)	11.78
Crude Protein, CP	14.70
Ca	0.52
P	0.32

^1^ Provided per kilogram of premix: VA—10,260 IU, VE—30 IU, VD3—2200 IU, Fe—57.86 mg, Zn—42.73 mg, Mn—33.65 mg, Cu—9.34 mg, Se—0.19 mg, I—0.76 mg, Co—0.23 mg.
