# Supplementary material for: Prolactin Inhibition Promotes Follicle Recruitment by Increasing PIKfyve Expression in Ewes During the Estrus Stage
Source: Animals (Basel). 2024 Dec 7;14(23):3541. doi: 10.3390/ani14233541 (PMC11639777; doi:10.3390/ani14233541)
Supplement: Supplementary file 1 [file animals-14-03541-s001.zip › animals-3317429-supplementary.pdf]

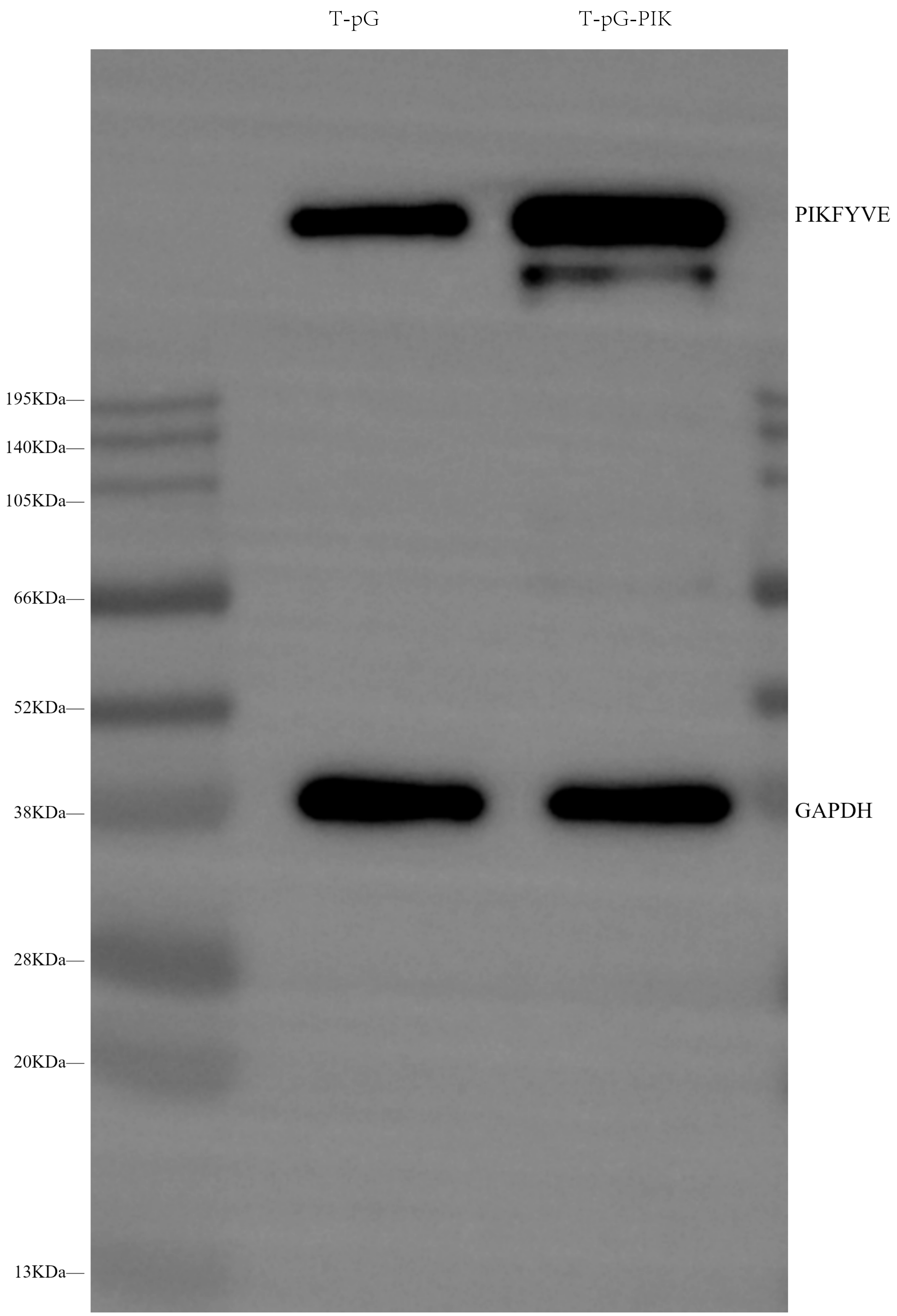
T-pG
T-pG-PIK
PIKFYVE
GAPDH
195KDa—
140KDa—
105KDa—
66KDa—
52KDa—
38KDa—
28KDa—
20KDa—
13KDa—

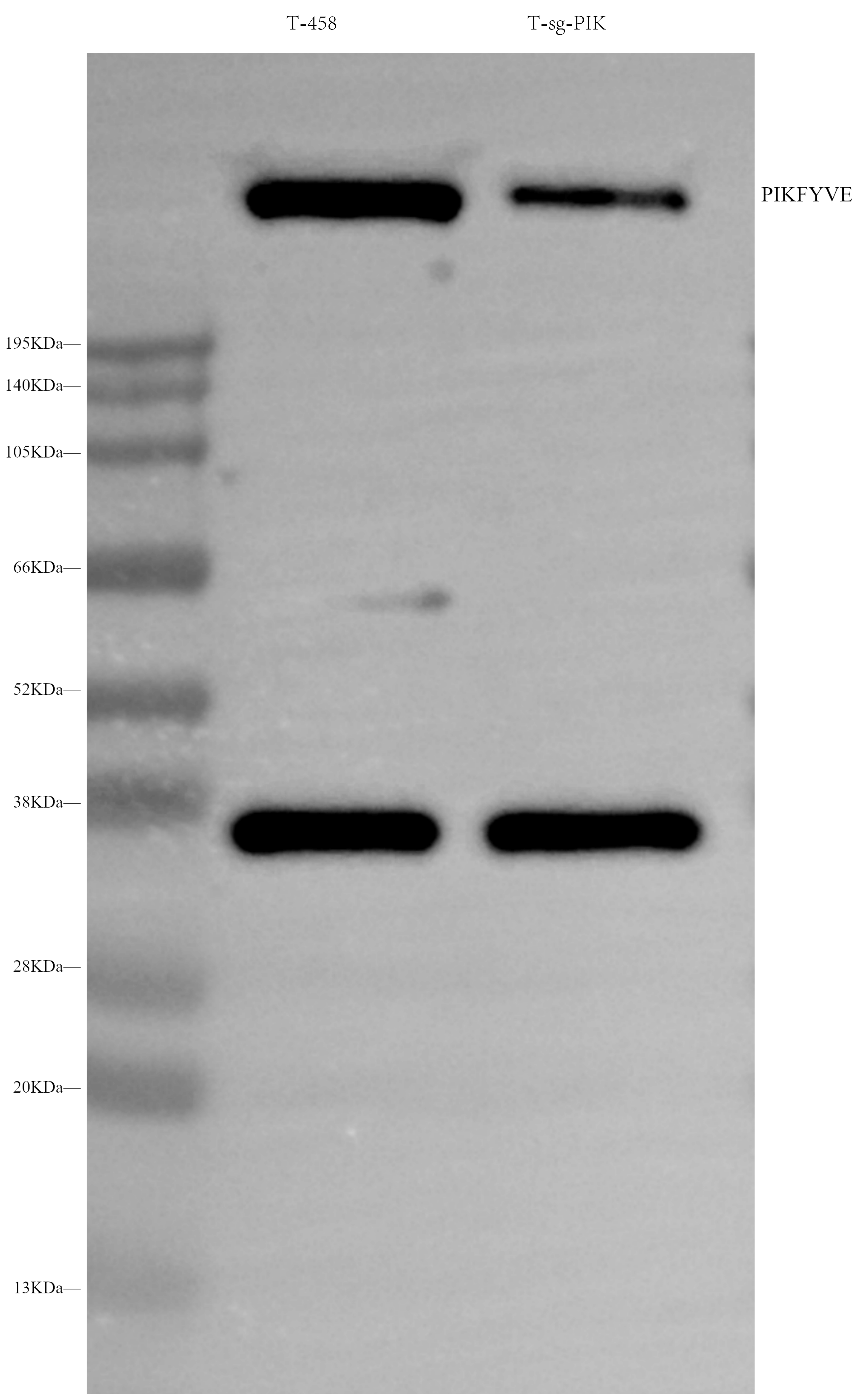
T-458
T-sg-PIK
PIKFYVE
195KDa—
140KDa—
105KDa—
66KDa—
52KDa—
38KDa—
28KDa—
20KDa—
13KDa—
